# Scrodentoids H and I, a Pair of Natural Epimerides from *Scrophularia dentata*, Inhibit Inflammation through JNK-STAT3 Axis in THP-1 Cells

**DOI:** 10.1155/2020/1842347

**Published:** 2020-07-27

**Authors:** Gaohui Mao, Liqin Sun, Jinwen Xu, Yiming Li, Ciren Dunzhu, Liuqiang Zhang, Fei Qian

**Affiliations:** ^1^Institute of Interdisciplinary Integrative Medicine Research, Shanghai University of Traditional Chinese Medicine, Shanghai 201203, China; ^2^School of Pharmacy, Shanghai University of Traditional Chinese Medicine, Shanghai 201203, China; ^3^Tibetan Traditional Medical College, Lhasa 850000, China

## Abstract

**Background:**

*Scrophularia dentata* is an important medicinal plant and used for the treatment of exanthema and fever in Traditional Tibetan Medicine. Scrodentoids H and I (SHI), a pair of epimerides of C_19_-norditerpenoids isolated from *Scrophularia dentata*, could transfer to each other in room temperature and were firstly reported in our previous work. Here, we first reported the anti-inflammatory effects of SHI on LPS-induced inflammation.

**Purpose:**

To evaluate the anti-inflammatory property of SHI, we investigated the effects of SHI on LPS-activated THP-1 cells.

**Methods:**

THP-1 human macrophages were pretreated with SHI and stimulated with LPS. Proinflammatory cytokines IL-1*β* and IL-6 were measured by RT-PCR and enzyme-linked immunosorbent assays (ELISA). The mechanism of action involving phosphorylation of ERK, JNK, P38, and STAT3 was measured by western Blot. The NF-*κ*B promoter activity was evaluated by Dual-Luciferase Reporter Assay System in TNF-*α* stimulated 293T cells.

**Results:**

SHI dose-dependently reduced the production of proinflammatory cytokines IL-1*β* and IL-6. The ability of SHI to reduce production of cytokines is associated with phosphorylation depress of JNK and STAT3 rather than p38, ERK, and NF-*κ*B promoter.

**Conclusions:**

Our experimental results indicated that anti-inflammatory effects of SHI exhibit attenuation of LPS-induced inflammation and inhibit activation through JNK/STAT3 pathway in macrophages. These results suggest that SHI might have a potential in treating inflammatory disease.

## 1. Introduction

Inflammation is a series of complex physiological reactions caused by persistent infection or dysregulation of immune responses in healthy tissues. It is an important process to remove exogenous stimuli and tissue damaged from the stimuli followed by initiating tissue repair, which is involved in the pathological processes of many diseases [1]. There are several immune cells involving inflammatory response, such as macrophages, neutrophils, and mononuclear phagocytes that secrete inflammatory mediators and inflammatory proteins upon stimulation by exogenous stimuli such as lipopolysaccharides (LPS) [2]. Macrophages are innate immune sentinels that patrol most tissues in the body. These cells detect changes in the microenvironment, including pathogen invasion and tissue damage, and mediate inflammatory processes, in response, that destroy microbial interlopers, remove and repair damaged tissue, and restore homeostasis [3]. Macrophages are versatile cells that orchestrate both the induction and the resolution of inflammation. Macrophages are crucial actors in innate immunity and modulating inflammatory response; therefore, activated macrophages are involved in the pathogenesis of multiple diseases. LPS is the major component of Gram-negative bacteria and one of the most potent inducers of inflammation. LPS can stimulate macrophages to release proinflammatory mediators and cytokines such as interleukin-6 (IL-6), tumor necrosis factor-*α* (TNF-*α*), interleukin-1 beta (IL-1*β*), activating mitogen-activated protein kinase (MAPK), nuclear factor-*κ*B (NF-*κ*B), and signal transducer and activator of transcription 3 (STAT3) pathway [4]. Toll-like receptor 4 (TLR4) activation caused by endotoxin with subsequent cytokine production is, in principle, beneficial for the organism, but when this process became dysregulated can lead to life-threatening syndromes [5, 6]. In the evolutionary context, control of overexuberant inflammation in order to maintain cell function is important to organism.

Pimarane diterpenoids are a complex group of plant-derived chemicals that have anti-inflammation [7, 8], anti-tumor [9–11], antimicrobial [12], vasorelaxant activity [13] and antifungal, antiviral, antispasmodic, and relaxant effects [14, 15]. Some of this type diterpenes exhibited dose-dependent inhibition of nitric oxide (NO) production [11, 16] and have been discovered as novel COX-2 inhibitors for the development of anti-inflammatory agents [17]. As a result of their efficacy in these settings, the search for new and biologically active pimarane diterpenoids has been on-going. Scrodentoids H and I (SHI) is a pair of epimerides of C_19_-norditerpenoids which belong to pimarane diterpenoids. They could transfer to each other in room temperature and the purified scrodentoid H or scrodentoid I could not be obtained. We firstly reported in our previous work [18]. SHI was isolated from *Scrophularia dentate* that is used for the treatment of exanthema and fever in Traditional Tibetan Medicine [18]. *S. dentata* has good effect on inflammation diseases. In our previous studies, we have reported some ingredient [19, 20] and pharmacology of this plant [21, 22]. SHI was an important diterpene isolated from this plant; however, the anti-inflammatory effect of SHI, as a novel natural compound, has not been researched.

In this study, we investigated the role of SHI in the LPS-stimulated inflammatory response through THP-1 cell model. THP-1, a human leukemia monocytic cell line, has been extensively modeled and used for investigating anti-inflammatory effects of compounds due to its unique characteristics [23]. The cells were usually stimulated with LPS, being in an activation state. We chose THP-1 as a model system because this cell line can be activated by LPS and can produce proinflammatory mediators such as TNF-*α*, IL-1*β*, and IL-6. In addition, it has been suggested that some diterpenes may have anti-inflammatory activity, particularly in LPS-induced macrophage responses [24–26]. Here, we found the anti-inflammatory effects of SHI on LPS-induced THP-1 cells, and it effectively inhibited these inflammatory through JNK-STAT3 axis.

## 2. Materials and Methods

### 2.1. Materials

THP-1 cells were purchased from the Chinese Academy of Sciences cell bank (Shanghai, China). 293T cells were saved in our group. LPS from *Escherichia coli* O111:B4 was purchased from Sigma Aldrich (St. Louis, MO, USA). Cell Proliferation Kit II was purchased from Roche (Germany). All PCR relative reagents were purchased from Thermo fisher (USA).

SHI was isolated and purified from *Scrophularia dentata* Royle ex Benth by our group as previous study [18], and the purity of SHI is more than 98%. SHI was dissolved in DMSO and stored in 50 mM concentration which was shown as the weight of SHI.

p-JNK, JNK, p-P38, P38, p-STAT3, and STAT3 antibodies were purchased from Cell Signaling Technology (USA). SP600125 inhibitor was purchased from Selleck Chemicals (USA). RIPA, BSA, PMSF, Nuclear and Cytoplasmic Protein Extraction Kit, and BCA Protein Assay Kit were purchased from Beyotime (China).

All chemicals were purchased from Sinopharm Chemical Reagent Co., Ltd., unless stated otherwise.

### 2.2. Cell Culture

THP-1 was cultured using Roswell Park Memorial Institute-1640 media (RPMI 1640) containing 10% fetal bovine serum (Gibco, Invitrogen, USA) and incubated at 37°C in 5% CO_2_/95% air. THP-1 cells were cultured in 100 mm dish and passage every three days. During experiments, the cells were plated in 96-well plates or 24-well plates.

### 2.3. Cell Viability

Cell viability was assessed using 2,3-bis[2-methoxy-4-nitro-5-sulfophenyl]-2H-tetrazolium-5-carboxanilide (XTT) assay. Cells were plated in 96-well plates at a density of 5 × 10^3^ cells/well and incubated with sample compounds. After culturing for 24 hours at 37°C, a solution of XTT and phenazine methosulfate (PMS) was added, and the culture was incubated for an additional 4 h. The absorbance at 492 nm and 690 nm was measured on a 96-well plate with a microplate reader. The percentage of cell viability was calculated as = (OD_test492_ − OD_test690_)/(OD_control492_ − OD_control690_) × 100%.

### 2.4. Quantitative Real-Time PCR

THP-1 cells were plated onto 24-well plates (2 × 10^6^ cells/well) and treated with different concentrations of compound SHI (10, 20, 40 and 60 *μ*M) or SP600125 for 1 h, followed by incubation with or without 1 *μ*g/ml LPS for 4 h. Then, total RNA was extracted from cells using Trizol. cDNA was synthesized from isolated total RNA using first strand cDNA synthesis kit (Thermo Scientific). Quantitative real-time PCR was carried out using gene specific primers (Table 1) and SYBR Green PCR Master Mix (Thermo Scientific) in a total volume of 20 *μ*L on Step One Plus Real-Time PCR System (Applied Biosystems). The relative expressions were expressed as fold change as determined by the relative quantification algorithm 2^−ΔΔCT^ Method.

### 2.5. Luciferase Assay

To assess NF-*κ*B promoter activity, 293T cells were transiently transfected with a luciferase reporter gene. Cells were plated one day prior to transfection, so that cells will be approximately 80% confluent on the day of transfection. On the day of transfection, DNA was diluted to 0.4 *μ*g per 60 *μ*L of serum-free medium, and an Attractene Transfection Reagent (QIAGEN, Germany) 1.5 *μ*L was added to achieve the proper ratio of reagent to DNA. The mixture was incubated for 20 minutes, and 60 *μ*L per DNA mixture was added to each well to be transfected. The effects of SHI on NF-*κ*B promoter activity were determined in 293T cells transfected with the pGL3-NF-*κ*B promoter luciferase construct, pRL-SV40 as an internal reference. Cells were transfected for 5 hours before changing to fresh media. One hour after changing to fresh media, 10 ng/mL TNF-*α* was added to the cells for 16 hours. Luciferase activity was measured in the cell lysates using the Dual-Luciferase Reporter Assay System according to the manufacturer's instructions (Promega, USA).

### 2.6. Enzyme-Linked Immunosorbent Assays (ELISA)

THP-1 cells at a density of 2 × 10^6^ cells/mL were incubated in 24-well plates with or without different concentrations of SHI for 1 hr and then induced with LPS (1 *μ*g/mL) for 24 hrs. Then, the supernatant was collected for measurement of cytokines. The concentrations of human IL-1*β* in culture supernatants were determined using the Duoset ELISA kits for cytokine (R&D Systems). The concentrations of cytokines IL-6 were measured using ELISA kits (invitrogen), according to the manufacturer's instructions.

### 2.7. Western Blot Analysis

THP-1 cells were pretreated with SHI for 1 hr and then stimulated with for 30 min or 4 hrs. After the treatments, cell lysates were prepared using radioimmunoprecipitation assay (RIPA) (Beyotime, China) and lysis buffer containing protease and phosphatase inhibitor cocktails. In another part of the experiment, cells were lysed in a Nuclear and Cytoplasmic Protein Extraction Kit (Beyotime, China) in order to obtain cytosol and nuclear fractions. The protein concentration was measured using the Bicinchoninic Acid (BCA) Protein Assay Kit (Beyotime, China). Equal concentrations of protein were separated using a 10% SDS-PAGE and transferred to PVDF membranes. After blocking, the membranes were incubated with antibodies against GAPDH, c-Jun N-terminal kinase (JNK), phospho-JNK, ERK, phospho-ERK, P38, phospho-P38, STAT3, phospho-STAT3 (Cell Signaling Technology, USA), and Lamin B (Santa Cruz Biotechnology, USA). The membranes were incubated with horseradish peroxidase- (HRP-) conjugated secondary antibodies (Cell Signaling Technology, USA) and developed using the enhanced chemiluminescence (ECL) detection system (Millipore, Eschborn, Germany).Protein activity was determined using a Chemiluminescent Imaging System (Tanon 5200 Multi) and Gel Image System (Tanon, China).

Protein was extracted for detecting p-ERK, ERK, p-P38, P38 p-JNK, JNK, and GAPDH antibodies for whole cell lysates and p-STAT3, STAT3, and Lamin B for nuclear fraction of THP-1 macrophages.

### 2.8. Statistical Analysis

All data were expressed as means ± SEM. Each value is the mean of three independent experiments. Statistical analysis was assessed via one-way ANOVA followed by Tukey post-hoc test by GraphPad Prism 6 (GraphPad, San Diego, CA, USA). A value of *P* < 0.05 was considered statistically significant.

## 3. Result

### 3.1. Cell Viability Validation of SHI

**Figure 1:**
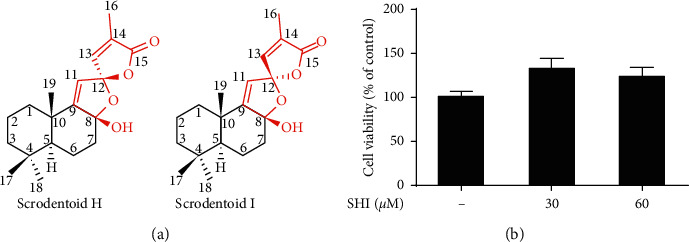
The structure of scrodentoids H and I and cell viability validation of SHI on THP-1 cells. (a) Chemical structures of scrodentoids H and I, a pair of natural epimerides from *Scrophularia dentata*. (b) THP-1 cells were treated with various concentrations of SHI (0∼60 *μ*M) for 24 h and then cell viability was confirmed by the XTT assay. All data are mean ± SEM, *n* = 3.

The chemical structures of SHI are shown in Figure 1(a). Before the anti-inflammation effect of SHI was measured, we referred to the testing concentration of compounds also from *Scrophularia dentata* Royle ex Bent [21, 22], and SHI was subjected to cytotoxic evaluation against THP-1 cell by XTT assay. After pretreatment with SHI treatment for 24 h, SHI did not find any cytotoxicity within 60 *μ*M in the XTT test of THP-1 cells (Figure 1(b)). Therefore, we used SHI at concentrations up to 60 *μ*M for all *in vitro* experiments.

### 3.2. SHI Inhibited IL-1*β* and IL-6 Production in THP-1 Cells

**Figure 2:**
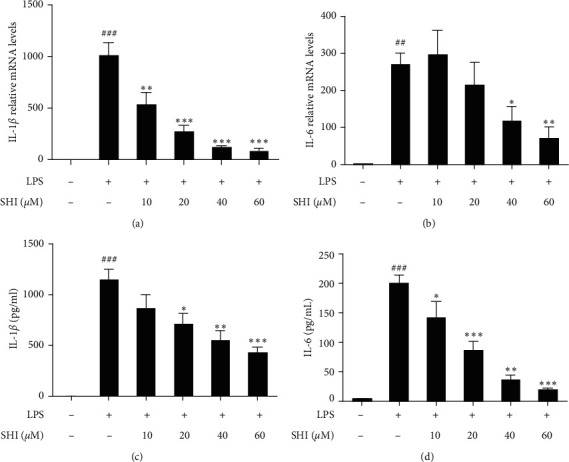
Scrodentoids H and I pretreatment reduced the secretion of proinflammatory cytokines and mRNA of IL-1*β* and IL-6 in LPS-induced THP-1 macrophages. (a, b) Cells were pretreated with various concentrations of scrodentoids H and I (0∼60 *μ*M) for 1 h following treatment of LPS (1 *μ*g/mL) for 4 h, and total RNA was extracted to detect relative mRNA levels. (c, d) Treatment with scrodentoids H and I for 1 h and then LPS stimulated 24 hours to collect cell supernatants for detection of various protein expressions. All data are mean ± SEM, *n* = 3, ^*∗*^*P* < 0.05,^*∗∗*^*P* < 0.01,^*∗∗∗*^*P* < 0.001 vs. LPS-stimulated cells and ^##^*P* < 0.01,^###^*P* < 0.001 vs. control.

To determine the anti-inflammatory effect of SHI, we examined the expression of IL-1*β* and IL-6 in LPS-induced THP-1 cells. We found that the mRNA levels of IL-1*β* (Figure 2(a)) and IL-6 (Figure 2(b)) were significantly increased after LPS-stimulated THP-1 cells for 4 h, and SHI significantly inhibited the expression of IL-1*β* and IL-6. Subsequently, we used ELISA to confirm the effect of SHI on the secretion of IL-1*β* and IL-6. As shown in Figures 2(c) and 2(d), the secretion of IL-1*β* (Figure 2(c)) and IL-6 (Figure 2(d)) exceeds 1000 pg/mL and 200 pg/mL, respectively. Pretreatment with SHI effectively inhibited the production of IL-1*β* and IL-6 in a concentration-dependent manner. Moreover, SHI at 60 *μ*M reduced the secretion of IL-6 up to 89.95%.

### 3.3. The Effect of SHI on NF-*κ*B Activity

**Figure 3:**
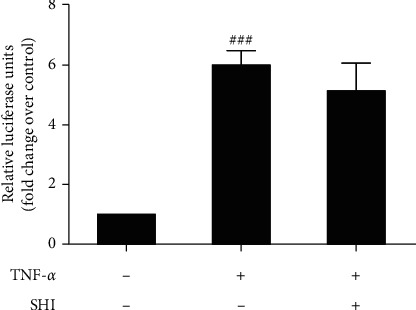
Scrodentoids H and I did not inhibit TNF-*α*-induced NF-*κ*B activation. 293T cells were preincubated with 50 *μ*M SHI for 1 h and then stimulated with 10 ng/mL TNF-*α* for 16 hours. The results shown are representative of three separate experiments. All data are mean ± SEM, *n* = 3, ^###^*P* < 0.001 vs. control.

Most of studies on terpenes have focused on NF-*κ*B pathway as a common target in explaining their anti-inflammatory and immunomodulatory effects [27, 28], particularly in pimarane diterpenes [29, 30]. Therefore, we explored whether SHI has effect on NF-*κ*B promoter activity or not. We used the Dual-Luciferase Reporter Assay System to detect luciferase activity of 293T cell lysates which has been transfected with the pGL3-NF-*κ*B promoter luciferase with SHI processing. However, SHI did not inhibit NF-*κ*B promoter activity (Figure 3). Thus, we speculate that the molecular mechanism of SHI on inflammation response might be not related to NF-*κ*B.

### 3.4. SHI Downregulated the Phosphorylation of STAT3

**Figure 4:**
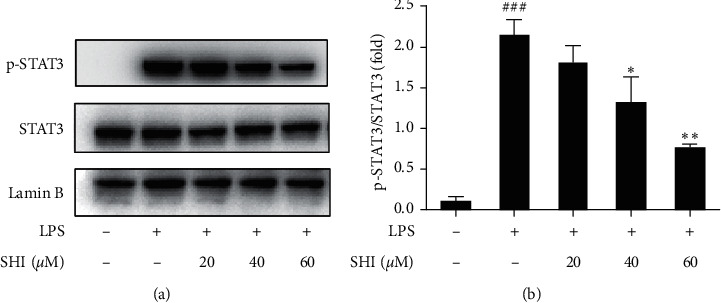
Scrodentoids H and I downregulated the phosphorylation of STAT3 induced by LPS in nuclear proteins. THP-1 cells were pretreated with various concentrations of SHI (0∼60 *μ*M) for 1 h following treatment of LPS (1 *μ*g/mL) for 4 h. (a) Nuclear proteins were extracted for western blot analysis to determine the expression levels of p-STAT3 and STAT3. (b) It is a statistical result of three independent experiments. All data are mean ± SEM, *n* = 3, ^*∗*^*P* < 0.05,^*∗∗*^*P* < 0.01 vs. LPS-stimulated cells and ^###^*P* < 0.001 vs. control.

Because IL-6 is mainly involved in the STAT pathway activation promoting acute and chronic inflammatory diseases and SHI has shown good IL-6 inhibitory activity, we examined the effect of SHI on the phosphorylation of STAT3. Cells were treated with LPS (1 *μ*g/ml) in the presence or absence of SHI for 4 h, and nuclear proteins were extracted for Western blot analysis to determine the expression levels of p-STAT3 and STAT3. As shown in Figure 4, after stimulation of LPS, p-STAT3 expression was significantly increased, and pretreatment with SHI considerably inhibited the phosphorylation of STAT3 in a dose-dependent manner.

### 3.5. SHI Decreased the Phosphorylation of JNK rather than ERK and P38

**Figure 5:**
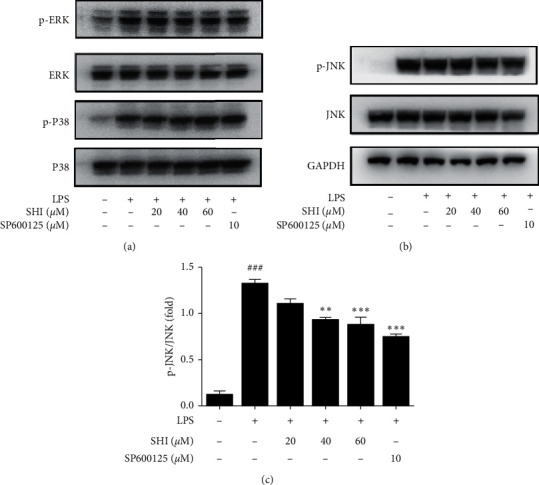
Scrodentoids H and I decreased the phosphorylation of JNK rather than ERK and P38. THP-1 cells were pretreated with various concentrations of SHI and SP600125 for 1 h following treatment of LPS (1 *μ*g/mL) for 30 mins. (a, b) Total proteins were extracted for western blot analysis to determine the expression levels of p-ERK, ERK, p-P38, P38, p-JNK, and JNK. (c) It is a statistical result of three independent experiments. All data are mean ± SEM, *n* = 3, ^*∗∗*^*P* < 0.01,^*∗∗∗*^*P* < 0.001, vs. LPS-stimulated cells and ^###^*P* < 0.001 vs. control.

MAPK signaling is another important pathway in inflammation response. Here, we tested the effect of SHI on the phosphorylation of ERK, P38, and JNK by western blot. We found that p-ERK, p-P38, and p-JNK were all increased after LPS was stimulated for 30 min in the THP-1 cell, but SHI did not alter the levels of p-ERK and p-P38 (Figure 5(a)). However, the upregulation of the phosphorylation of JNK was significantly attenuated following pretreatment with SHI (Figures 5(b) and 5(c)).

### 3.6. SP600125 Reduced the Phosphorylation of STAT3

The aforementioned data showed that p-JNK increased after LPS stimulation for 30 min in THP-1 cells, and p-STAT3 was changed after 4 h of LPS stimulation. However, no report about the relationship between JNK and STAT3 in the THP-1 cells was found. Here, we hypothesized that JNK might regulate the activity of STAT3, thereby promoting the release of inflammatory mediators in THP-1 cells.

**Figure 6:**
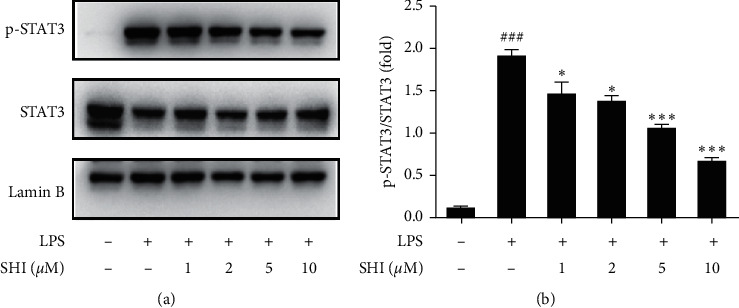
Inhibition of JNK pathway with SP600125 reduced the p-STAT3 gene expression in nuclear proteins. Cells were pretreated with various concentrations of SP600125 (0∼10 *μ*M) for 1 h following treatment of LPS (1 *μ*g/mL) for 4 h. (a) Nuclear proteins were extracted for western blot analysis to determine the expression levels of p-STAT3 and STAT3. (b) It is a statistical result of three independent experiments. All data are mean ± SEM, *n* = 3, ^*∗∗*^*P* < 0.01,^*∗∗∗*^*P* < 0.001, vs. LPS-stimulated cells and ^###^*P* < 0.001 vs. control.

To further elucidate the mechanism of SHI in LPS-stimulated THP-1 cells and the above hypothesis, the phosphorylation of STAT3 was measured by western blot utilizing JNK inhibitor SP600125. Data showed that SP600125 significantly inhibited the phosphorylation of STAT3 in THP-1 cells (Figures 6(a) and 6(b)). These results implied that LPS-mediated STAT3 signal transduction in THP-1 cells may be regulated by JNK pathways.

### 3.7. The Effect of SP600125 on Cytokines Production in LPS-Induced THP-1 Cells

**Figure 7:**
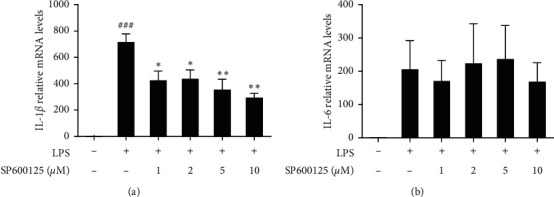
The effect of SP600125 on mRNA of IL-1*β* and IL-6. (a, b) THP-1 cells were pretreated with various concentrations of SP600125 (0∼10 *μ*M) for 1 h following treatment of LPS (1 *μ*g/mL) for 4 h, and total RNA was extracted to detect relative mRNA levels. All data are mean ± SEM, *n* = 3, ^*∗*^*P* < 0.05,^*∗∗*^*P* < 0.01 vs. LPS-stimulated cells and ^###^*P* < 0.001 vs. control.

To further confirm the relationship between the anti-inflammation effect of SHI and JNK signaling pathway, we investigated the effect of JNK- inhibition on IL-1*β* and IL-6 mRNA levels. Thus, THP-1 cells were pretreated with SHI for 1 h, followed by LPS stimulated for 4 h. As shown in Figure 7, SP600125 suppressed LPS-induced IL-1*β* expression in a dose-dependent manner in THP-1 cells (Figure 7(a)). However, SP600125 was not reduced IL-6 mRNA levels (Figure 7(b)). Therefore, these results indicated that IL-6 is upstream of STAT3, and it might be stimulated by autocrine.

## 4. Discussion

*Scrophularia dentata* is used for the treatment of exanthema and fever in Traditional Tibetan Medicine. SHI is an important diterpene isolated from this plant; however, SHI as a novel natural compound has not been researched. In this study, we first reported its anti-inflammatory effects on LPS-induced THP-1 cells via JNK/STAT3 axis.

Inflammation is a part of the immune response against injury or pathogenic infection. Infection or cell damage triggers the release of proinflammatory mediators which play critical roles in the pathogenesis of many acute and chronic diseases. IL-1*β*, a classic proinflammatory mediator, plays a key role in septic shock, rheumatoid arthritis, inflammatory bowel disease, and type II diabetes and is thus a major therapeutic target [31]. IL-6 is a pleiotropic cytokine that plays an important role in immune and inflammatory responses. In our research, we found that pretreatment with SHI reduced both the mRNA expression and release of IL-1*β* and IL-6 in LPS-induced THP-1 macrophages (Figure 2).

Signal transduction of proinflammatory cytokines includes many pathways, such as NF-*κ*B, MAPK, and STAT signaling pathway [32]. For example, MAPKs critically contribute to initiate the activation of transcription factor and produce proinflammatory mediators and cytokines and are considered as therapeutic targets of inflammatory diseases [33], especially as JNK. For example, Chrysin could inhibit the release of IL-1*β* via inhibiting the activation of JNK [34]. In addition, JNK activation in the LPS-induced expression of IL-6 in neutrophils has been described. Inhibition of JNK by its inhibitor SP600125 decreased IL-6 mRNA expression in LPS-stimulated neutrophils in a dose-dependent manner [35]. Also, some data suggest that inhibition of LPS-induced IL-6 production is caused by inhibiting the JNK signaling pathway and activation of AP-1 in microglia [36]. Similarly, it is shown that lower IL-6 and IL-1*β* were conducive to protect against lethality in sepsis syndrome via the JNK signaling pathway and the transcription factor AP-1 [37]. Therefore, we investigated the involvement of SHI in MAPK activation. Interestingly, SHI significantly suppressed LPS-induced activation of JNK 1/2 (Figures 5(b) and 5(c)) but did not alter the phosphorylation of p38 MAPK and ERK 1/2 (Figure 5(a)). In addition, we measured the effect of SHI on NF-*κ*B which is a key regulator of inflammation; however, SHI did not reduce the increase of NF-*κ*B promoter activity in THP-1 cells (Figure 3).

STAT proteins play a central role in regulating cytokine-dependent inflammation [38–40]. 7 STATs (STAT1, STAT2, STAT3, STAT4, STAT5a, STAT5b, and STAT6) are identified and STAT3 is highly associated with inflammatory response. In response to inflammatory stress, STAT3 directly serves as a transcription factor to regulate expression of proinflammatory cytokines [41]. STAT3 activation has been widely reported to maintain epithelial homeostasis and cytokine balance [42, 43]. Therefore, we investigated the effect of SHI on phosphorylation of STAT3. SHI significantly suppressed the phosphorylation of STAT3 in LPS-induced THP-1 cells (Figure 4). Furthermore, we tried to detect the changes of p-JAK2 and JAK2 which is a classic protein that could active the phosphorylation of STAT3. Under our experimental conditions, however, the phosphorylation of LPS-stimulated JAK2 protein was at the edge of the lowest detection limit, and there was no statistical difference between treatment with LPS and no treatment in THP-1 cells (Figures S1 and S2). It is indicated that SHI in THP-1 cells regulates the activation of STAT3 by other pathways.

Above all, the molecular mechanism of SHI is mainly related to JNK and STAT3 pathway. Previous literature report has reported that JNK was located upstream of STAT3 in some types of cells. For example, a study showed that inhibition of JNK reduced G alpha(s)-mediated STAT3 phosphorylation [44]. Furthermore, G proteins have been implicated in TLR4 signaling in macrophages. In response to LPS, G proteins subunit alpha-1 and alpha-3 form complexes containing the pattern recognition receptor CD14, which are required for activation of PI3K-Akt signaling. G proteins deficiency decreased LPS-induced TLR4 endocytosis. These data confirm the importance of G proteins in TLR4-mediated responses in cells and reveal a level of TLR and signaling pathway specificity [45, 46]. The above two sections explain the relationship between the LPS/TLR4- G alpha(s)-JNK- STAT3 axis. However, the relationship between JNK and STAT3 has not been reported in THP-1 cells. Here, we wondered if there was a critical link between the JNK and STAT3 in THP-1 cells. As expected, we found that the phosphorylation level of STAT3 was also regulated by the JNK inhibitor SP600125 (Figure 6), suggesting that inhibition of JNK could affect the activity of STAT3. However, SP600125 suppressed LPS-induced IL-1*β* expression without altered IL-6 expression (Figure 7). Thus, we hypothesize that the effects of SHI on JNK and STAT3 were produced after LPS stimulation for 30 min and 4 h, respectively, and that other proteins should play a role in this interval. Some literatures have shown that LPS/TRL4 signaling can also produce inflammatory cytokines via the oxidative stress-PYK2-STAT3 pathway [47–49]. In addition, a study showed that Pyk2 activated p-JNK and then promoted proinflammatory mediators release in human aortic endothelial cells [50]. Meanwhile, some literatures have reported that PYK2 could regulate the release of IL-6 in different diseases such as cancer and inflammation related diseases [51, 52]. Thus, we speculate that PYK2 may be the key to the tandem action of JNK and STAT3, and this hypothesis remains to be verified experimentally.

Activating STAT3 via G proteins in THP-1 cells by CCR1 agonist, leukotactin-1 (CCL15) could induce IL-6 and IL-8 production. Neutralizing antibody to IL-6 inhibited CCL15-mediated STAT3 Tyr705 phosphorylation, whereas inhibition of STAT3 activity abolished CCL15-activated IL-8 release [53]. IL-6 plus its soluble receptor sIL-6R (IL-6/sIL-6R) promoted THP-1 monocyte migration, and statins blocked IL-6/sIL-6R-induced translocation of STAT3 to the nucleus by inhibiting JAK/STAT signaling cascades [54]. Blocking IL-6/STAT3 signaling defers inflammation responses central to the progression of diabetic nephropathy [55] and atherogenic responses [56]. Moreover, STAT3 inhibition could represent a possible future therapeutic target in systemic lupus erythematosus [57]. However, activation of STAT3 has the capability to drive hepatocyte compensatory proliferation, a key principle of the regenerating liver [58]. Simultaneously, cardiac STAT3 is important for maintaining metabolic homeostasis, and loss of STAT3 in cardiomyocytes makes the heart more susceptible to chronic pathological insult [59]. In recent years, a plethora of studies have convincingly shown that only signaling via the soluble IL-6R (transsignaling) accounts for the deleterious effects of IL-6, whereas classic signaling via the membrane-bound receptor is essential for the regenerative and antibacterial effects of IL-6 (classic signaling) [60]. We also noticed the double-edged effect of STAT3 activation in the muscles, and STAT3 is a critical regulator of satellite cell self-renewal after muscle injury. However, prolonged STAT3 activation in muscles has been shown to be responsible for muscle wasting [61]. STAT3 in various cell types of the gut may individually contribute to the restoration of intestinal homeostasis on the one hand or pave the way for excessive immunopathology on the other, as an inflammatory “rheo-STAT” [62]. STAT3 can have a plethora of effects in inflammation-related diseases. It is important to balance the extent of STAT3 activation and the duration and location (cell types) of the STAT3 signaling when developing therapeutic interventions.

## 5. Conclusions

In the present study, we firstly demonstrated the anti-inflammatory effects of SHI by attenuating LPS-induced inflammation and inhibiting the JNK/STAT3 pathway in macrophages. Our results suggest SHI, a natural diterpene compound isolated from *Scrophularia dentata* Royle ex Benth, might have benefit for treating inflammatory-related diseases such as ulcerative colitis and atherosclerotic disease.

## Figures and Tables

**Table 1:** Primer sequences used in real-time PCR.

Gene	Forward primer (5′-3′)	Reverse primer (5′-3′)
IL-1*β*	TGAAATGATGGCTTATTACAGTGGC	GTAGTGGTGGTCGGAGATTCGTAG
IL-6	CCTCCAGAACAGATTTGAGAGTAGT	GGGTCAGGGGTGGTTATTGC
GAPDH	CGCTGAGTACGTCGTGGAGTC	GCTGATGATCTTGAGGCTGTTGTC

## Data Availability

The data used to support the findings of this study are available from the corresponding author upon request.

## Abbreviations

LPS: Lipopolysaccharide
IL-6: Interleukin-6
TNF-*α*: Tumor necrosis factor-*α*
IL-1*β*: Interleukin-1 beta
TLR4: Toll-like receptor 4
COX-2: Cyclooxygenase-2
NO: Nitric oxide
DMSO: Dimethyl sulfoxide
ECL: Enhanced chemiluminescence
FBS: Fetal bovine serum
HRP: Horseradish peroxidase
IL: Interleukin
JNK: c-Jun *N*-terminal kinase
MAPK: Mitogen-activated protein kinase
RIPA: Radioimmunoprecipitation assay
RT-PCR: Reverse transcription-polymerase chain reaction
NF-*κ*B: Nuclear factor-*κ*B
STAT3: Signal transducer and activator of transcription 3
PYK2: Proline-rich tyrosine kinase 2.

## Supplementary Materials

Supplementary Materials After LPS stimulation, the phosphorylation of JAK2 was at the edge of the lowest detection limit, and there was no statistical difference between treatment with LPS or no treatment in THP-1 cells.
