# Supplementary material for: Scrodentoids H and I, a Pair of Natural Epimerides from Scrophularia dentata, Inhibit Inflammation through JNK-STAT3 Axis in THP-1 Cells
Source: Evid Based Complement Alternat Med. 2020 Jul 27;2020:1842347. doi: 10.1155/2020/1842347 (PMC7403932; doi:10.1155/2020/1842347)

Supplementary Information

Scrodentoids H&I, a pair of natural epimerides from *Scrophularia dentata*, inhibits inflammation through JNK-STAT3 axis in THP-1 cells

Gaohui Mao[1,2], Liqin Sun[1,2], Jinwen Xu[1], Yiming Li[2], Ciren Dunzhu[3], Liuqiang Zhang[2]*, Fei Qian[1]*

Email: 04100217@163.com (L.Z.); qianfei0517@126.com (F. Q.)

This PDF file includes:

Supplementary text

Figures. S1, S2

SI Materials and Methods

Materials

p-JAK2 antibody waspurchased from Abcam, JAk2 antibody was purchased from Cell Signaling Technology(USA). CST PC was a cell extract which is a positive control of p - JAK2 from CST Company.

Method

THP-1 cells were pretreated with SHI for 1 hr, and then stimulated with for 30 min. After the treatments, cell lysates were prepared using radio-immunoprecipitation assay (RIPA) (Beyotime, China) lysis buffer containing protease and phosphatase inhibitor cocktails. The protein concentration was measured using the bicinchoninic acid (BCA) protein assay kit (Beyotime, China). Equal concentrations of protein were separated using a 10% SDS-PAGE and transferred to PVDF membranes. After blocking, the membranes were incubated with antibodies against p-JAK2, JAK2, GAPDH. The membranes were incubated with horseradish peroxidase (HRP-conjugated secondary antibodies (Cell Signaling Technology, USA) and developed using the enhanced chemiluminescence (ECL) detection system (Millpore, Eschborn, Germany) USA).Protein activity was determined using a Chemiluminescent Im aging System (Tanon 5200 Multi) and Gel Image System (Tanon, China).

SI Figure legends

Figure. S1 The effect of SHI and SP600125 on p-JAK2/JAK2. Cells were pre-treated with various concentrations of SHI or SP600125 for 1 h following treatment of LPS (1 μg/mL) for 30 min. Figure A, B, C are presented three independent experiments.

Figure. S2 The effect of CST PC and our samples on p-JAK2. CST PC was a positive control cell extracts from CST Company. Another four samples, THP-1 cells were pre-treated without or with SP600125 for 1 h following treatment of LPS (1

µg/mL) for 30 min.

Figures

Figure S1

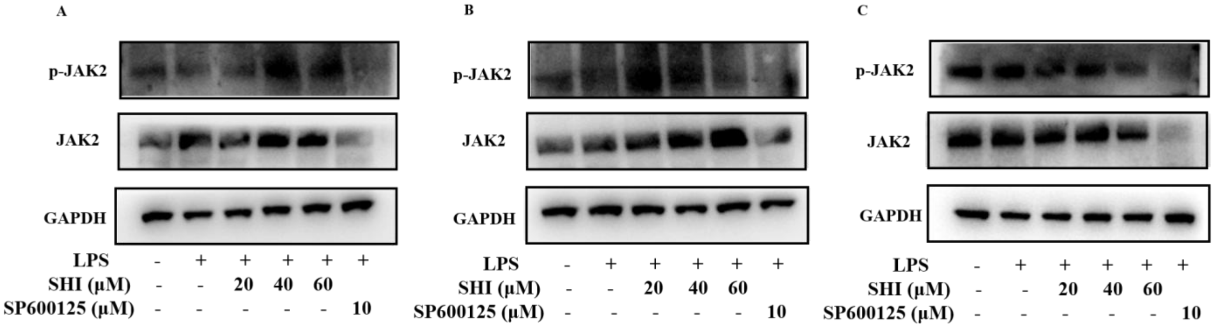

Figure S2

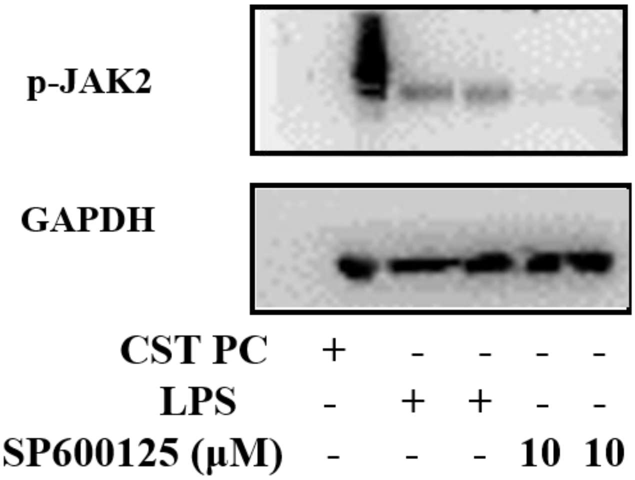

Supplement: Supplementary Materials — After LPS stimulation, the phosphorylation of JAK2 was at the edge of the lowest detection limit, and there was no statistical difference between treatment with LPS or no treatment in THP-1 cells. [file 1842347.f1.pdf]
